# Tumor-priming CD8^+^ natural killer T-like cells as an efficient novel cell therapy for relapsed/refractory multiple myeloma

**DOI:** 10.1186/s40164-025-00707-7

**Published:** 2025-09-29

**Authors:** Juheon Lee, Eunjeong Choi, Bohwa Han, Jeong-a Kim, Dana Jung, Kyeong-Hee Kim, Sung Yong Oh, Sung-Hyun Kim, Kyung-Soo Ha, Ji-Hoon Kim, Ji Hyun Lee, Duck cho, Junsang Doh, Seok-Ho Kim

**Affiliations:** 1 Department of Health Sciences, The Graduate School of Dong-A University, Busan, 49315 Republic of Korea; 2 Department of Materials Science and Engineering, Seoul National University, 1 Gwanak-ro, Gwanak-gu, Seoul, 08826 Republic of Korea; 3 Department of Medicinal Biotechnology, College of Health science, Dong-A University, Busan, 49315 Republic of Korea; 4 Department of Laboratory Medicine, Dong-A University College of Medicine, Busan, Republic of Korea; 5 Division of Hematology-Oncology, Department of Internal Medicine, Dong-A University College of Medicine, Busan, Republic of Korea; 6 Department of Laboratory Medicine and Genetics, Samsung Medical Center, Sungkyunkwan University School of Medicine, 81, Irwon‑Ro, Gangnam‑Gu, Seoul, 06351 Republic of Korea; 7 Osong Medical Innovation Foundation, 123 Osongsaengmyung-ro, Osong-eup , Chungbuk, Heungduk-gu, Chungju-si, Republic of Korea

**Keywords:** Cellular immunotherapy, Relapsed/refractory multiple myeloma, NKT-like cells, Hematologic malignancies

## Abstract

**Background:**

Relapsed and refractory multiple myeloma (RRMM) remains a major clinical challenge, as most patients eventually relapse following standard treatments and are left with limited therapeutic options. Although b-cell maturation antigen (BCMA) CAR-T cell therapy has recently shown remarkable efficacy in select patients, broader implementation is hindered by its reliance on autologous cells, prolonged manufacturing timelines, high costs, and severe immune-related toxicities. These challenges have prompted an urgent demand for safer, more accessible, and rapidly applicable immunotherapeutic alternatives.

**Methods:**

CBMC (cord blood mononuclear cells) were cultured with irradiated BMMC (bone marrow mononuclear cells) from RRMM patients in the presence of defined cytokines, aiming to develop a new therapeutic immune cell product for RRMM. Their phenotypic and functional characteristics, including non-MHC-restricted and MHC-restricted cytotoxicity mechanisms, were analyzed using surface marker profiling, cytokine secretion assays, in vitro cytotoxicity assays, functional and blocking assays. Antitumor activity was evaluated in xenograft mouse models using MM.1 S and RPMI-8226 cells.

**Results:**

We successfully generated CD8^+^ NKT-like cells through tumor priming, which exhibited potent cytotoxicity and elevated cytokine production against multiple myeloma cell lines and primary RRMM samples. Mechanistically, tumor-priming CD8^+^ NKT-like cells (TPNC) cytotoxicity was mediated by both non-MHC–restricted pathways involving LFA-1 and DNAM-1, and MHC-restricted, TCR-mediated recognition. TPNC efficiently formed immune synapses, rapidly polarized cytotoxic granules, and engaged in serial killing. In xenograft models, TPNC significantly suppressed tumor progression, prolonged survival, and persisted in circulation without observable toxicity. Based on these findings, we extended the tumor-priming strategy to acute myeloid leukemia (AML) and acute lymphoblastic leukemia (ALL), successfully generating TPNC with robust cytotoxic activity. In ALL samples, TPNC exhibited cytotoxicity comparable to anti-CD19 CAR-NK cells.

**Conclusions:**

TPNC represents a novel cytotoxic lymphocyte product generated through tumor-driven priming. Their dual recognition capacity, functional versatility, and favorable safety profile highlight their potential as a scalable and personalized immunotherapy platform for hematologic malignancies.

**Supplementary Information:**

The online version contains supplementary material available at 10.1186/s40164-025-00707-7.

## Introduction

Multiple myeloma (MM) is a malignant plasma cell disorder that, despite significant therapeutic advances, remains largely incurable [1, 2]. Standard treatments, including proteasome inhibitors, immunomodulatory agents, monoclonal antibodies, and autologous stem cell transplantation, have improved patient outcomes, but most individuals eventually relapse or develop treatment resistance [3–5]. In the relapsed/refractory multiple myeloma (RRMM), the median overall survival remains poor, typically ranging from 2 to 3 years in heavily pretreated populations [6–8].

B-cell maturation antigen (BCMA)–targeted CAR-T cell therapies, such as idecabtagene vicleucel and ciltacabtagene autoleucel, have demonstrated high response rates in RRMM, including deep remissions and minimal residual disease (MRD) negativity [9–11]. However, their long-term efficacy is limited, with over half of patients relapsing within 1–2 years [12, 13]. Moreover, CAR-T therapies are frequently associated with severe immune-related toxicities, such as cytokine release syndrome (CRS) and immune effector cell–associated neurotoxicity syndrome (ICANS), and their autologous nature results in prolonged manufacturing timelines and restricted accessibility, particularly in rapidly progressing disease [14–16]. These limitations underscore the need for safer, more scalable, and readily deployable immune cell therapies.

Recent studies have shown that innate lymphocytes, particularly NK cells, can acquire memory-like features upon chronic antigen stimulation, including enhanced cytotoxicity, proinflammatory cytokine production, and long-term persistence [17–19]. These adaptive NK cells exhibit clonal-like expansion, epigenetic remodeling, and improved recall responses following repeated exposure to viral or tumor-associated antigens [20, 21]. Their superior functional profile and persistence have made them promising candidates for next-generation cancer immunotherapy.

Based on this finding, we aimed to generate highly cytotoxic, memory-like NK cells through a tumor-priming strategy using umbilical cord blood mononuclear cells (CBMC) co-cultured with irradiated, patient-derived RRMM cells under defined cytokine conditions. Contrary to our expectations, the culture system predominantly yielded CD8⁺ NKT-like cells, rather than memory-like NK cells. To understand the identity and function of this unexpected population, we conducted comprehensive phenotypic and functional analyses. The tumor-priming CD8^+^ NKT-like cells (TPNC) exhibited potent cytotoxicity against myeloma cell lines and patient-derived RRMM samples, robust secretion of IFN-γ and TNF-α, and efficient immune synapse formation with polarized granule release. Mechanistically, they employed both MHC-restricted and MHC-independent cytolytic pathways, consistent with a hybrid innate-adaptive identity.

In xenograft mouse models, TPNC significantly suppressed tumor progression, prolonged survival, and persisted in vivo without observable toxicity. These findings support the potential of TPNC as a non-genetically modified, scalable, and effective immunotherapeutic platform for RRMM and other hematologic malignancies.

## Materials and methods

### Human materials and isolation

This study was approved by the Institutional Review Board of Dong-A University (approval number: 2-1040709-AB-N-01-202303-BR-002-02) and the ethics committee of the participating center (DAUHIRB-23-029) in accordance with the Declaration of Helsinki. All human participants provided written informed consent. Bone marrow samples were collected during routine clinical procedures to assess the status of clonal plasma cells in patients with RRMM, and leukemic cells in patients with acute lymphocytic leukemia (ALL) or acute myeloid leukemia (AML). CBMC and BMMC were isolated from the leukocyte layer by density gradient centrifugation using Lymphoprep™ (STEMCELL Technologies, Canada). Clinical information for RRMM patients is summarized in Supplementary Table S1, and data for ALL and AML patients are provided in Supplementary Table S2.

### CIK cells and TPNC culture

Both CIK cells and TPNC were generated from CBMC isolated by milling and density gradient centrifugation. Cells were cultured in CTS™ NK-Xpander™ medium (Gibco, USA) supplemented with 5% human platelet lysate (HPL; DYNE BIO, Korea) and 1% penicillin/streptomycin (Capricorn, Germany) in a humidified incubator at 37 °C with 5% CO₂.

#### CIK cells culture

A total of 3 × 10⁶ CBMC were seeded into 24-well plates. On day 0, 1000 U/mL recombinant human IFN-γ (Peprotech, USA) was added. On day 1, cells were treated with 50 ng/mL anti-CD3 antibody (Invitrogen, USA) and 20 ng/mL IL-2 (Peprotech). Culture medium was refreshed every 2–3 days with fresh medium containing 20 ng/mL IL-2.

#### TPNC culture

To generate TPNC, 3 × 10⁶ CBMC were co-cultured with 5 × 10⁵ X-ray–irradiated patient-derived feeder cells (BMMC from RRMM, ALL, or AML patients) in 24-well plates. Feeder cells were irradiated to prevent their proliferation and eliminate the risk of viable tumor cell contamination during the co-culture process, as commonly performed in cell expansion protocols. On day 0, cells were stimulated with 20 ng/mL IL-2 and 5 ng/mL IL-21 (PeproTech), and 25 ng/mL IL-18 (R&D Systems). On days 3 and 5, the medium was refreshed with IL-2 (20 ng/mL). On day 7, 1 × 10⁶ TPNC were re-stimulated with 5 × 10⁵ irradiated BMMC in fresh medium containing 20 ng/mL IL-2 and 5 ng/mL IL-21, which was replenished every 2–3 days thereafter.

### Cell lines

Human-derived multiple myeloma (IM-9 and RPMI-8226), ALL (CCRF-CEM and MOLT4), and AML (HL-60 and THP-1) cell lines were purchased from the Korean Cell Line Bank. Cells were maintained in RPMI 1640 medium (Welgene, Korea) supplemented with 10% Fetal Bovine Serum (FBS; Welgene, Korea) and 1% penicillin/streptomycin (Capricorn, Germany). The U266 and MM.1 S cell lines expressing firefly luciferase (MM.1 S-luc) was kindly provided by Professor Sunghoon Jung (Chonnam National University Hwasun Hospital, Korea). All cell lines were routinely tested for mycoplasma contamination.

### Flow cytometry

Flow cytometry was performed to monitor dynamic changes in CD3 and CD56 expression during cell culture and to evaluate the phenotype and cytotoxic molecule expression of CIK cells and TPNC. For surface marker analysis, cells were collected at 7-day intervals from day 0 to day 21 and stained with antibodies against CD3 and CD56. For endpoint analysis, 1 × 10⁵ cells were washed, incubated with antibodies for 30 min at 4 °C in the dark, and analyzed by flow cytometry. For intracellular staining, cells were first labeled with surface antibodies, then fixed and permeabilized using a commercial fixation/permeabilization buffer set. Subsequently, cells were stained for perforin, and granzyme B under dark conditions at 4 °C. Stained samples were acquired using an Attune NxT flow cytometer (Thermo Fisher Scientific, USA), and data were analyzed using FlowJo software version 10.9.0 (FlowJo, LLC, USA). Antibody details are listed in Supplementary Table S3. All phenotypic analyses were gated on the CD3⁺CD56⁺ population unless otherwise specified.

### Cytotoxicity assay

To assess cytotoxic activity, CIK cells and TPNC were used as effector cells, while human MM (IM-9, RPMI-8226, U266), ALL (CCRF-CEM, MOLT4), AML (HL-60, THP-1) cell lines, and primary samples from RRMM, ALL, and AML patients were used as target cells. All target cells were labeled with carboxyfluorescein succinimidyl ester (CFSE), and co-cultured with effector cells in 96-well plates at various effector-to-target (E: T) ratios for 4 h at 37 °C in 5% CO₂. After co-culture, cells were stained with fixable viability dye (FVD) to detect cell death. Cytotoxicity was analyzed using an Attune NxT flow cytometer (Thermo Fisher Scientific, USA) and FlowJo v10.9.0 software (FlowJo, LLC, USA). Target cell killing was quantified as the percentage of CFSE⁺FVD⁺ cells.

### Degranulation assay

Degranulation was assessed by measuring CD107a surface expression. Effector and target cells were co-cultured at a 1:1 effector-to-target (E: T) ratio in 96-well plates in the presence of anti-CD107a antibody and GolgiStop (Monensin; BD Biosciences, USA) for 4 h at 37 °C in 5% CO₂. After incubation, cells were stained with antibodies against CD3 and CD56 to identify effector cells. Subsequently, cells were fixed and permeabilized using Cytofix/Cytoperm and washed with Perm/Wash buffer (BD Biosciences, USA). CD107a expression was analyzed in the CD3⁺CD56⁺ population using an Attune NxT flow cytometer (Thermo Fisher Scientific, USA), and data were processed with FlowJo software v10.9.0 (FlowJo, LLC, USA).

### ELISA

CIK cells and TPNC were co-cultured with target cells at a 1:1 effector-to-target (E: T) ratio in 96-well plates for 24 h. Culture supernatants were collected and analyzed for human IFN-γ and TNF-α levels using ELISA kits (Abcam, UK) according to the manufacturer’s instructions.

### TCR sequencing and analysis

Total RNA was extracted from TPNC samples and used for TCRβ library construction. First-strand cDNA was synthesized using a TCR-specific reverse primer, followed by two rounds of PCR: a multiplex PCR (PCR-1) using Vβ-specific primers containing UMIs and constant region-specific primers, and a second PCR (PCR-2) to add dual indices and Illumina adapters. Final libraries were quantified by qPCR, and fragment sizes were assessed using Agilent Tapestation. Sequencing was performed on an Illumina NovaSeq X Plus platform with 150 bp paired-end reads. Raw reads were merged with ≥ 60 bp overlap, and UMI-based error correction was applied. Sequences were aligned to the NCBI IgBLAST TR gene database using IgBLAST (v3.6) with filters for in-frame VJ rearrangements and quality thresholds (V identity ≥ 80%, J identity ≥ 80%). Clustering was performed with CD-HIT at 95% identity. Representative clonotypes were selected by abundance, and clone counts were normalized and visualized using VDJtools and Immunarch. CDR3 amino acid sequences were annotated via the VDJdb database.

### Live cell imaging

Cells were prepared as described in the Supplementary Materials and Methods (Supplementary Information). A modified Olympus IX 83 epifluorescence microscope equipped with a 40 × (UPlanFLN, NA = 1.30) objective and an ANDOR Zyla 4.2 sCOMS camera were used for the imaging experiments. The microscope was automatically controlled using Micro-manager.

### Animal studies

Female NOD-Prkdc ^em1Baek^ Il2rg ^em1Baek^ (NSGA) mice (6- weeks-old) were obtained from GEM Biosciences Inc. (Cheongju, Republic of Korea). All animal procedures and maintenance were approved by the Institutional Animal Care and Use Committee (DIACUC-23-49). For the disseminated tumor model, 5 × 10^6^ MM.1 S-luciferase human multiple myeloma cells were intravenously injected into NSGA mice (*n* = 6 per group). On day 5 post-tumor injection, 5 × 10⁶ CIK cells or TPNC in 100 µL PBS were administered intravenously. The control group received an equal volume of PBS. Tumor progression was monitored weekly via bioluminescence imaging using the IVIS Spectrum system (Xenogen, Caliper Life Sciences, USA), and total flux (photons/sec) was quantified. At the experimental endpoint (day 40), peripheral blood was collected, and the persistence of human immune cells was analyzed by flow cytometry. For the subcutaneous tumor model, 1 × 10⁶ RPMI-8226 cells were injected into the flank of NSGA mice (*n* = 5 per group). On day 7, 5 × 10⁶ CIK cells or TPNC were injected intravenously. Tumor volume was measured twice weekly using calipers and calculated as (width)² × length × 0.5. Mice were sacrificed on day 21 for tumor weight analysis and ex vivo tumor imaging. For survival analysis, NSGA mice were intravenously injected with 1 × 10⁶ RPMI-8226 cells and monitored until the defined humane endpoints were reached. Survival was analyzed using the Kaplan–Meier method. All mice were housed in the Dong-A University Animal Facility and all experiments were performed following the relevant institutional and national guidelines.

### Statistical analysis

All experiments were independently repeated at least three times to ensure reproducibility. Statistical comparisons between two groups were performed using a two-tailed unpaired Student’s t-test. For comparisons among three or more groups, one-way ANOVA followed by appropriate post hoc tests was used. Survival data were analyzed using Kaplan–Meier survival curves. All statistical analyses were conducted using GraphPad Prism 9 software (GraphPad Software, USA). P-values < 0.05 were considered statistically significant. The specific statistical tests used for each experiment are indicated in the respective Figure and Figure Legends.

## Results

### Generation of tumor-priming CD8^+^ NKT-like cells (TPNC) to target patient-derived RRMM cells

To develop an effective cellular immunotherapy for RRMM, we initially investigated the cytotoxic capacity of cord blood–derived NK (CB-NK) cells. CB-NK cells exhibited potent cytotoxicity against established myeloma cell lines such as IM-9 and RPMI-8226, confirming their baseline antitumor activity. However, when tested against primary tumor cells isolated from RRMM patients, CB-NK cells displayed markedly diminished cytotoxicity (Fig. S1A and B). This observation highlights a critical translational gap between preclinical efficacy in cell lines and therapeutic relevance in patient-derived tumor models, limiting the utility of conventional NK cell approaches for RRMM. To address this limitation, we established a tumor-priming strategy utilizing patient-derived tumor cells to induce memory-like NK cells with enhanced antitumor properties. To this end, CBMC were co-cultured with irradiated (50 Gy) BMMC from RRMM patients in the presence of a defined cytokine cocktail.

**Fig. 1:**
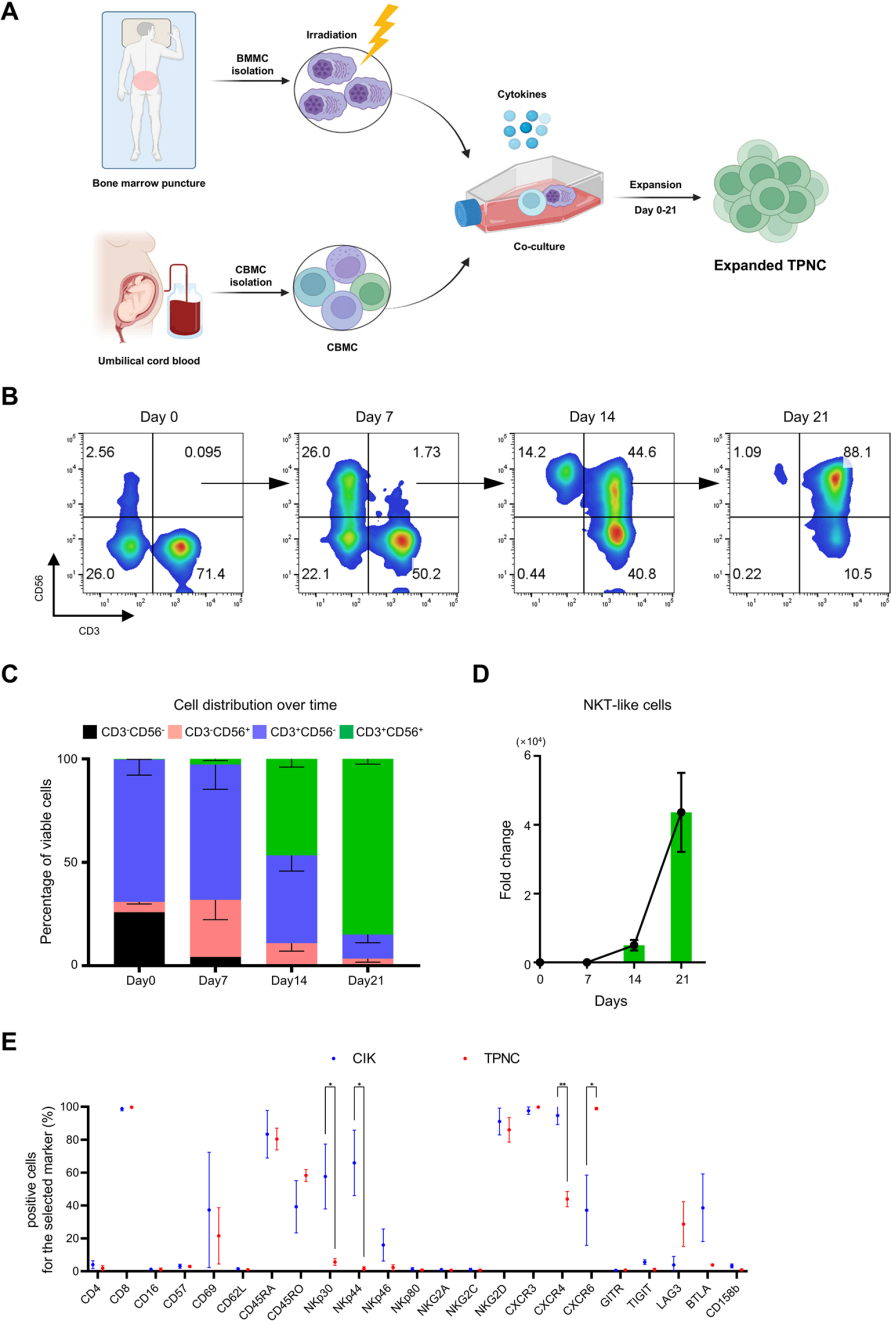
Generation and phenotypic characterization of tumor-priming CD8⁺ NKT-like cells (TPNC). **A** Schematic of TPNC generation using co-culture of CBMC and X-irradiated (50 Gy) BMMC from RRMM patients. **B** Representative flow cytometry plots showing the distribution of CD3⁻CD56⁻, CD3⁻CD56⁺, CD3⁺CD56⁻, and CD3⁺CD56⁺ cells over 21 days of culture. **C** Quantification of the four cell subsets shown in (**B**). **D** Fold expansion of CD3⁺CD56⁺ NKT-like cells, calculated by dividing the absolute number at each time point by the number at day 0. **E** Surface expression of selected markers on TPNC and conventional CIK cells analyzed by flow cytometry. Data are presented as mean ± SD of at least three independent experiments. *P < 0.05; and **P < 0.01 based on Student’s t-test

On day 0, CBMC were co-cultured with BMMC derived from RRMM patients, in the presence of IL-2 (20 ng/mL), IL-18 (25 ng/mL), and IL-21 (5 ng/mL). Additional IL-2 (20 ng/mL) was supplemented on days 3 and 5. On day 7, the same patient-derived BMMC were added, and the culture was maintained for 21 days with continuous supplementation of IL-2 and IL-21 (Fig. 1A). Unexpectedly, under these conditions, a heterogeneous population of immune cells was generated instead of the initially intended cytotoxic NK cell subset (Fig. S2A). Among these, CD3⁺CD56⁺ NKT-like cells selectively expanded, comprising approximately 85% of the total cell population by day 21, with a mean fold expansion of ~ 43,000 (Fig. 1B–D). Functional analysis revealed that NKT-like cells exhibited stronger cytotoxic activity than NK cells and CD3⁺ T cells isolated from the same culture (Fig. S2B), suggesting that tumor-priming selectively enhanced the cytotoxic potential of the NKT-like subset.

To further characterize these NKT-like cells, we performed immunophenotypic profiling based on surface receptor expression. CD3⁺CD56⁺ cytokine-induced killer (CIK) cells were used as a reference population. While both cell types expressed similar lineage and activation markers, moderate differences were observed in the expression of NKp30, NKp44, CXCR4, and CXCR6 (Fig. 1E).

Collectively, these findings indicate that although the culture system was originally designed to expand cytotoxic NK cells, tumor-priming CD8^+^ NKT-like cells (TPNC) emerged as the dominant population.

### Enhanced cytotoxicity and functional activity of TPNC against RRMM cells in vitro

**Fig. 2:**
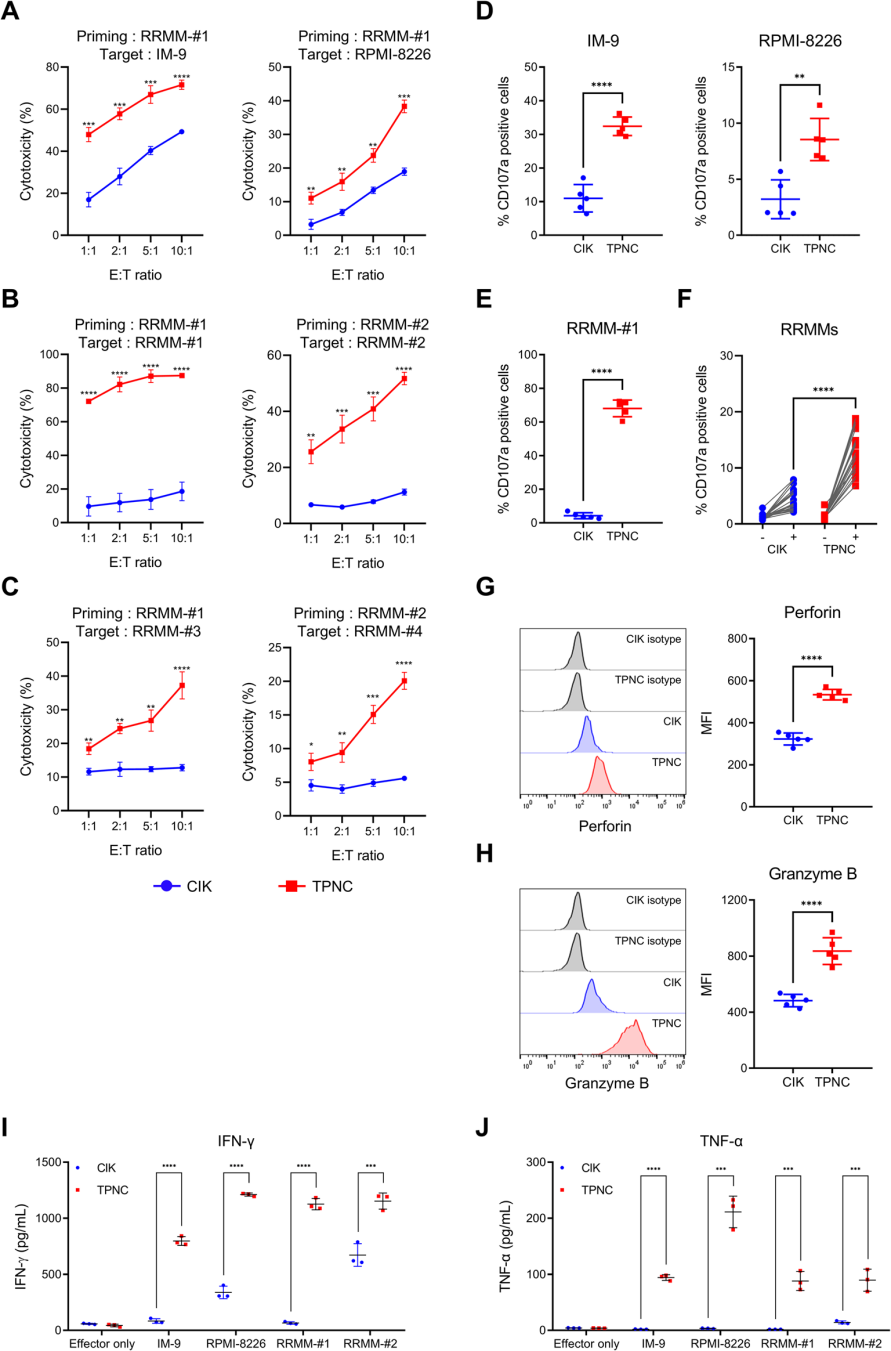
Functional cytotoxicity and immune responses of TPNC in vitro. **A–C** Cytotoxicity of TPNC and CIK at various effector-to-target (E: T) ratios against MM cell lines **(A)**, priming-matched RRMM cells (**B**), and RRMM cells from patients not used in the priming process (**C**). **D–F** Degranulation measured by CD107a expression after co-culture with MM cell lines (**D**), RRMM-#1 (**E**), and RRMM samples from eight patients not used in priming **(F)**. **G–H** Intracellular levels of perforin (**G**) and granzyme B (**H**) in TPNC and CIK assessed by flow cytometry. **I–J** IFN-γ (**I**) and TNF-α (**J**) production in response to MM cell lines and RRMM cells, measured by ELISA. Data are presented as mean ± SD of at least three independent experiments. **P < 0.01; ***P < 0.001; and ****P < 0.0001 based on Student’s t-test

To evaluate the cytotoxic potential of TPNC, we assessed their activity against both MM cell lines and patient-derived RRMM cells. TPNC exhibited significantly enhanced cytotoxicity across various effector-to-target (E: T) ratios, including IM-9 and RPMI-8226 (Fig. 2A), autologous RRMM samples used during priming (RRMM #1 and #2; Fig. 2B), and additional unrelated patient-derived RRMM cells (RRMM #3 and #4; Fig. 2C).

In addition, TPNC showed increased surface expression of CD107a and higher intracellular levels of perforin and granzyme B compared to CIK cells, indicating enhanced degranulation capacity (Fig. 2D–H). Furthermore, TPNC secreted significantly higher levels of IFN-γ and TNF-α than CIK cells in response to stimulation with MM cell lines and primary RRMM cells. (Fig. 2I and J).

Taken together, these results demonstrate that TPNC has superior effector functions compared to CIK cells, characterized by enhanced cytotoxic activity, robust degranulation, and elevated production of proinflammatory cytokines in response to both MM cell lines and patient-derived RRMM cells.

### TPNC utilizes both non-MHC and MHC-restricted cytotoxicity mechanisms

To investigate the mechanisms underlying TPNC-mediated cytotoxicity, we initially focused on non–MHC-restricted pathways, which are well characterized in CIK cell [22]. NKG2D, a key activating receptor associated with MHC-independent killing, was expressed at comparable levels in both TPNC and CIK cells (Fig. 1E). However, MM cell lines and patient-derived RRMM samples exhibited low expression of NKG2D ligands (Fig. S3), suggesting that the NKG2D axis plays a limited role in this system.

**Fig. 3:**
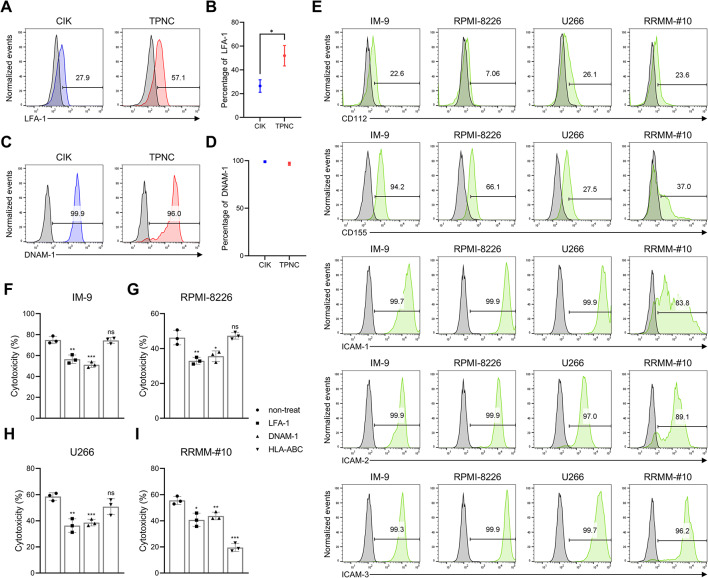
Mechanistic investigation of TPNC-mediated killing. **A–D** Flow cytometry analysis of LFA-1 and DNAM-1 expression in CIK and TPNC. **E** Surface expression of their corresponding ligands—CD112, CD155, and ICAM-1/2/3—on MM cell lines (IM-9, RPMI-8226, U266) and a primary RRMM sample (RRMM-#10). **F–H** Cytotoxicity of TPNC against MM cell lines following antibody-mediated blockade of LFA-1, DNAM-1, or HLA-ABC. **I** Cytotoxicity against the priming-matched RRMM-#10 under the same blockade conditions. Data are presented as mean ± SD of at least three independent experiments. *P < 0.05; **P < 0.01; and ***P < 0.001 based on Student’s t-test

We therefore examined additional non–MHC-restricted pathways involving LFA-1 and DNAM-1 [21, 23, 24], which are also implicated in CIK-mediated cytotoxicity [25]. TPNC expressed significantly higher levels of LFA-1 than CIK cells, while DNAM-1 was abundantly expressed in both populations (Fig. 3A–D). Their corresponding ligands, CD112, and CD155, were broadly expressed on MM cell lines and patient-derived RRMM cells (Fig. 3E). Functionally, blocking either LFA-1 or DNAM-1 significantly reduced TPNC-mediated cytotoxicity against multiple MM cell lines (IM-9, RPMI8226, U266) as well as the autologous RRMM-#10 sample (Fig. 3F–I), confirming that these non–MHC-restricted pathways contribute substantially to TPNC cytotoxicity.

Meanwhile, since NKT-like cells have been reported to mediate MHC-restricted cytotoxicity [26, 27], we next assessed the involvement of this pathway using blocking experiments. HLA-ABC blockade had no effect on TPNC-mediated killing of MM cell lines (Fig. 3F–H), but significantly reduced cytotoxicity against the priming-matched RRMM-#10 sample (Fig. 3I). Consistent results were also observed in additional autologous samples (RRMM-#8 and #9; Fig. S4A), supporting the possibility that TPNC may engage in antigen-specific, TCR-mediated cytotoxicity, particularly in autologous settings.

To further clarify whether MHC-restricted cytotoxicity is limited to autologous targets or extends to unrelated patient samples within the same malignancy, we performed HLA-ABC blocking experiments using TPNC primed with RRMM-#10 against unrelated RRMM-#8 and RRMM-#9 targets. HLA-ABC blockade led to a significant reduction in cytotoxicity against these unrelated RRMM targets (Fig. S4B), indicating that TPNC-mediated killing can also be MHC class I–dependent even in the context of unrelated patient-derived tumor cells of the same disease subtype.

Taken together, these results indicate that TPNC primarily rely on non–MHCrestricted mechanisms involving LFA-1 and DNAM-1, but can also utilize MHCrestricted cytotoxicity in an antigendependent manner. Notably, in three independent donor–target combinations (patients #8, #9, and #10), HLAI blockade reduced killing even against unrelated RRMM samples, suggesting the presence of shared tumorassociated antigens within this malignancy subtype. Further validation in a broader panel of RRMM models will clarify the generality of this mechanism.

### TCR repertoire remodeling during TPNC expansion reflects antigen-driven selection mediated by apcs in CBMC

**Fig. 4:**
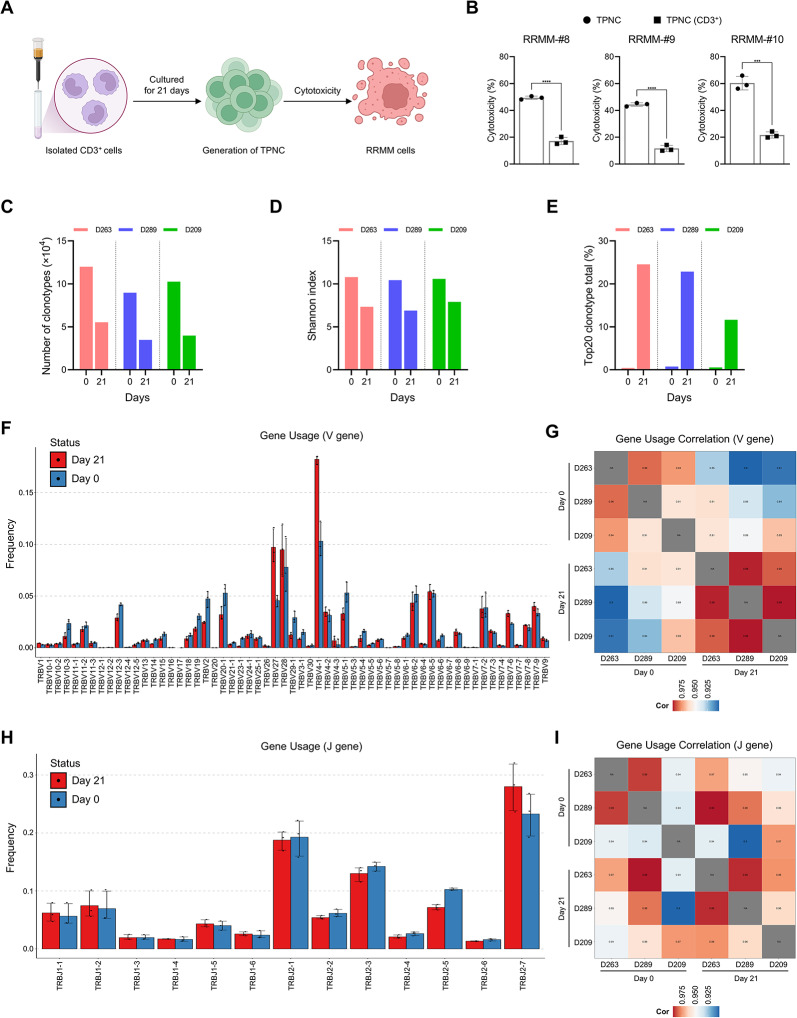
TCR clonal selection during TPNC expansion is driven by APCs in CBMC. **A** Schematic of the experimental setup to generate TPNC with or without APCs by using isolated CD3⁺ cells. **B** Cytotoxicity of standard TPNC versus CD3⁺-only derived TPNC against three primary RRMM samples at an E: T ratio of 5:1, demonstrating reduced function in the absence of APCs. **C-E** TCR repertoire analysis showing decreased clonotype diversity and increased clonal dominance in TPNC on day 21, based on clonotype number (**C**), Shannon diversity index (**D**), and top 20 clonotype frequency (**E**). **F** Bar graph showing the relative frequency of TRBV gene usage across three donors on day 0 and day 21. **G** Heatmap displaying pairwise similarity in TRBV usage, with clustering based on sampling timepoints. **H** Bar graph showing the relative frequency of TRBJ gene usage across three donors on day 0 and day 21. **I** Heatmap displaying correlation of TRBJ usage across samples, grouped by day 0 and day 21. Data are presented as mean ± SD of at least three independent experiments. ***P < 0.001; and ****P < 0.0001 based on Student’s t-test

Given that APCs were present and activated within the CBMC culture system (Fig. S5A–C), we hypothesized that these cells might facilitate MHC-dependent priming during TPNC generation. To evaluate the contribution of APCs to TPNC functional programming, CD3⁺ T cells were isolated from CBMC and cultured under identical priming conditions in the absence of APCs (Fig. 4A). Under these APCs-depleted conditions, TPNC exhibited markedly reduced cytotoxicity against patient-derived RRMM cells (Fig. 4B), indicating that APCs are essential for the acquisition of cytotoxic function likely via TCR-dependent antigen priming.

To further investigate whether clonal selection occurs during the priming phase, we performed TCR repertoire sequencing on TPNC derived from three independent CBMC donors. At day 21, TPNC showed a significant reduction in clonotype diversity compared to day 0 CBMC, including fewer unique clonotypes, decreased Shannon diversity, and dominance of select TCR clonotypes (Fig. 4C–E), suggesting antigen-driven clonal expansion. Analysis of V(D)J gene usage revealed consistent enrichment of TRBV4-1, TRBV27, TRBV28, and TRBJ2-7 in day 21 samples (Fig. 4F–I), further supporting the presence of antigen-specific TCR remodeling during the priming process.

Although the specific antigens remain unidentified, these findings collectively suggest that APCs within the CBMC population may present tumor-derived antigens via MHC class I, enabling antigen-specific programming and expansion of CD8⁺ NKT-like cells during TPNC generation.

### Efficient immune synapse formation contributes to the rapid killing activity of TPNC

**Fig. 5:**
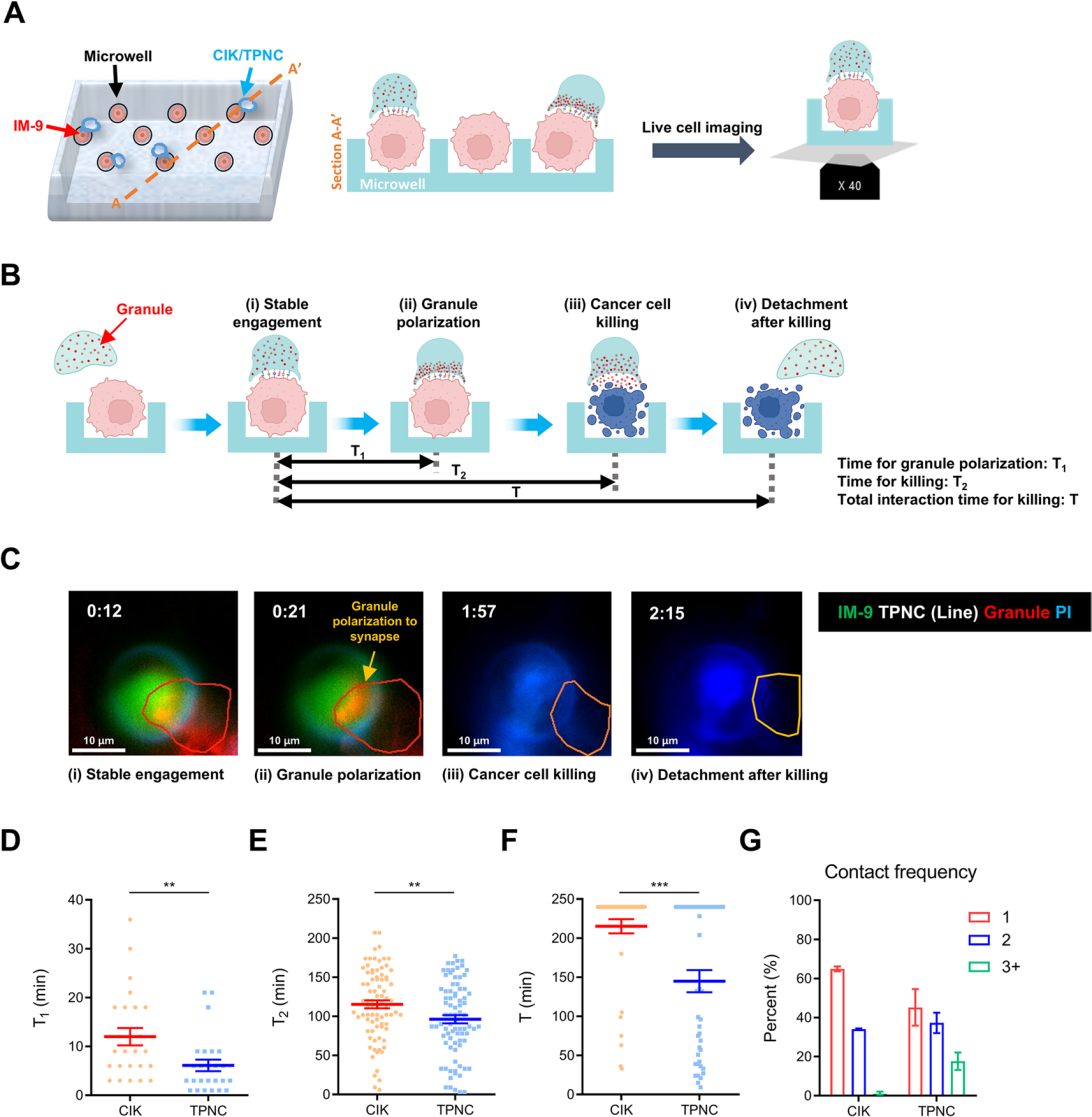
Live-cell imaging reveals efficient immune synapse formation and rapid killing by TPNC. **A** Schematic of single cell cytotoxicity assay using a microwell array. IM-9 cells (red) were seeded into individual wells, and CIK or TPNC cells (blue) were added to visualize immune synapse formation. **B** Sequential stages of effector–target interaction: stable engagement, granule polarization, target cell killing, and detachment. T_1_ represents the time to granule polarization, T_2_ the time to killing, and T the total interaction time. **C** A representative time-lapse imaging of a TPNC killing an IM-9 cell. Time stamp: hh: mm. Green: IM-9; red: granule; blue: PI; orange line: effector cells (CIK and TPNC). **D–F** Quantification of granule polarization time (T_1_), killing time (T_2_), and total interaction time (T). **G** Contact frequency showing the number of target cells contacted per effector cell during 5 h of imaging. Data are presented as mean ± SD of at least three independent experiments. **P < 0.01; and ***P < 0.001; based on Student’s t-test

The increased expression of LFA-1 (Fig. 3A), a key molecule involved in immune synapse formation [28, 29], suggested that enhanced synapse formation might contribute to the cytotoxic function of TPNC. To investigate this hypothesis, we performed live-cell imaging to visualize and quantify the immune synapse dynamics between TPNC or CIK cells and MM targets at the single-cell level. IM-9 cells were seeded into microwell arrays and co-cultured with either TPNC or CIK cells (Fig. 5A). Target cells were labeled with CFSE, and lytic granules within effector cells were stained with LysoTracker. We tracked four sequential steps in the killing process: (i) stable engagement, (ii) granule polarization, (iii) target cell lysis, and (iv) detachment (Fig. 5B). Representative time-lapse images demonstrated each step of the TPNC-mediated killing process (Fig. 5C), and a corresponding video is provided (Movie S1).

Quantitative analysis revealed that TPNC formed immune synapses more rapidly and efficiently than CIK cells. Specifically, TPNC required shorter time intervals to achieve granule polarization (T_1_), target cell lysis (T_2_), and total engagement duration (T) (Fig. 5D–F). Moreover, TPNC displayed superior serial killing capacity, frequently engaging and lysing multiple tumor targets in succession, unlike CIK cells (Fig. 5G).

Collectively, these findings indicate that TPNC form more efficient immune synapses than CIK cells, enabling rapid cytotoxic responses and enhanced serial killing of tumor cells.

### TPNC demonstrates potent in vivo antitumor activity and exhibits minimal toxicity

**Fig. 6:**
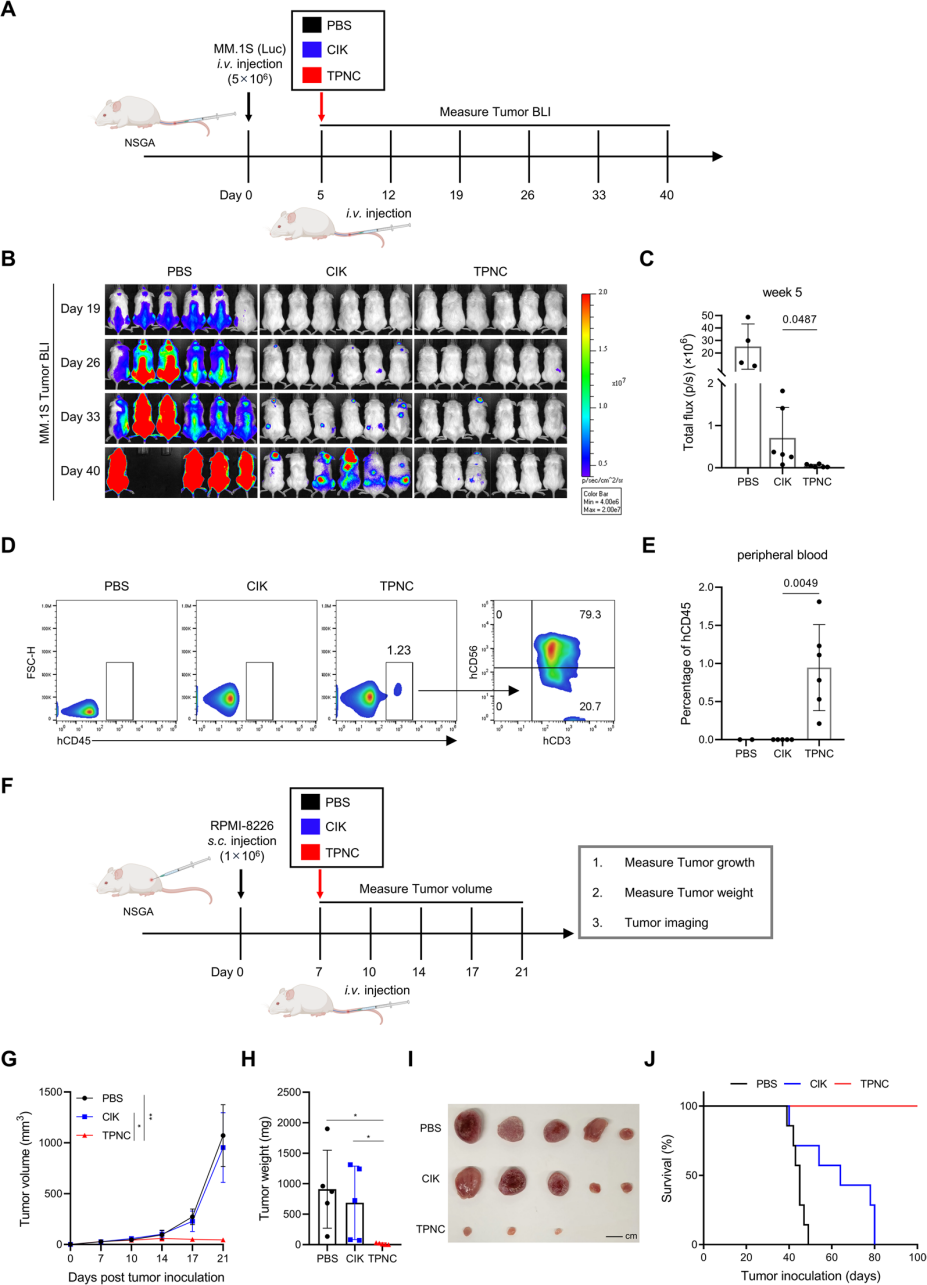
In vivo efficacy and persistence of TPNC in mouse models of multiple myeloma. **A** Schematic of systemic MM model using luciferase-expressing MM.1 S cells intravenously injected into NSGA mice on day 0, followed by adoptive transfer of PBS, CIK, or TPNC on day 5. **B** Bioluminescence imaging (BLI) of tumor burden at indicated time points (days 19, 26, 33, and 40). **C** Quantification of total tumor flux at week 5. **D** Representative flow cytometry plots showing human CD45⁺ cells in peripheral blood on day 40. Only TPNC-treated mice exhibited detectable human cells, which were further analyzed for CD3 and CD56 expression. **E** Frequency of human CD45⁺ cells in peripheral blood across treatment groups. **F** Schematic of subcutaneous tumor model using RPMI-8226 cells. Tumors were established via subcutaneous injection on day 0, followed by cell therapy on day 7. **G** Tumor volume was monitored over time. **H** Tumor weight was measured at endpoint (day 21). **I** Representative images of excised tumors from each treatment group. **J** Kaplan–Meier survival analysis of mice intravenously injected with RPMI-8226 cells and treated with PBS, CIK, or TPNC. Data are presented as mean ± SD of at least three independent experiments. *P < 0.05; and **P < 0.01 based on one-way ANOVA followed by post hoc tests

To evaluate the in vivo therapeutic potential of TPNC, we employed both disseminated and localized MM models in NSG mice. In the disseminated model, luciferase-expressing MM.1 S cells were intravenously injected, followed by adoptive transfer of TPNC or control cells on day 5 (Fig. 6A). TPNC-treated mice exhibited significantly reduced tumor burden compared to PBS and CIK groups, as measured by bioluminescence imaging (Fig. 6B and C). Notably, TPNC were still detectable in peripheral blood at week 5, with higher frequencies than control cells, indicating superior in vivo persistence (Fig. 6D and E). To further assess systemic anti-tumor activity, we generated a subcutaneous xenograft model using RPMI-8226 cells. Seven days after tumor implantation, TPNC were administered intravenously, resulting in significant suppression of tumor growth and reduced final tumor weights compared to PBS- and CIK-treated groups (Fig. 6F–H). Notably, complete tumor regression was observed in two mice (Fig. 6I), and Kaplan–Meier survival analysis further demonstrated that TPNC treatment significantly prolonged overall survival (Fig. 6J).

Together, these findings demonstrate that TPNC possess potent in vivo antitumor activity and durable persistence in preclinical MM models, supporting their translational potential as an allogeneic immunotherapy. To assess the safety of TPNC as an allogeneic cell therapy candidate, we evaluated the potential for off-target immune responses. NSG mice were intravenously administered 1 × 10⁸ TPNC or CIK cells once weekly for three consecutive weeks and monitored over an 8-week period. No significant changes in body weight were observed (Fig. S6A), and histopathological analysis of major organs revealed no signs of tissue damage or inflammation (Fig. S6B), indicating the absence of GvHD (Graft-versus-host disease)-like symptoms.

To further examine potential off-target cytotoxicity, TPNC were tested against autologous PBMC from the same donor and allogeneic CBMC. No cytotoxic effects were observed against either healthy cell population (Fig. S6C and D).

Together, these findings indicate that TPNC selectively eliminate malignant cells without inducing immune responses against normal immune cells, supporting their safety and potential utility as a scalable allogeneic immunotherapy product.

### TPNC demonstrates potent cytotoxicity against diverse hematologic malignancies

**Fig. 7:**
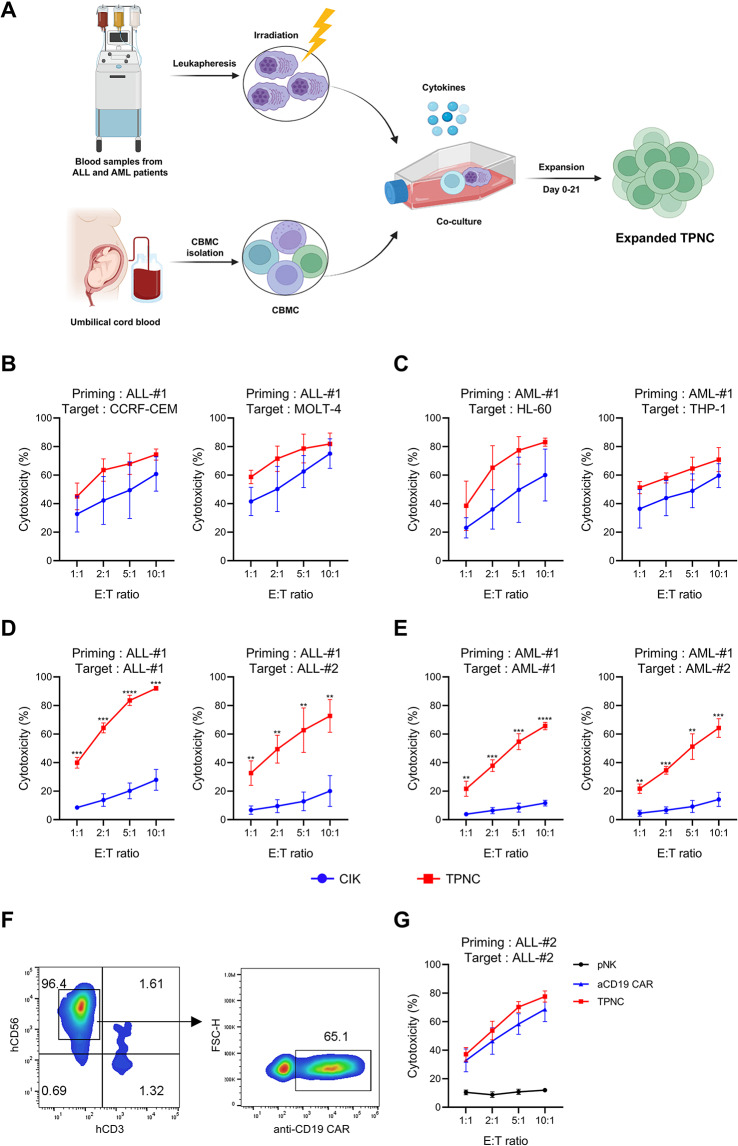
Cytotoxicity of TPNC against leukemia cell lines and primary samples from ALL and AML patients. **A** Schematic of TPNC generation using co-culture of CBMC and leukapheresis samples from patients with ALL and AML. **B–C** Cytotoxicity of TPNC and CIK against leukemia cell lines including CCRF-CEM, MOLT-4 **B**, and HL-60, THP-1 **C**. **D–E** Cytotoxicity of TPNC and CIK against primary leukemia cells from ALL patients (**D**) and AML patients **(E)**, including samples from both the priming donor and other patients not used during priming. **F–G** Comparison of TPNC with pNK and anti-CD19 CAR-NK cells in cytotoxicity assays against primary ALL cells. Data are presented as mean ± SD of at least three independent experiments. **P < 0.01; ***P < 0.001; and ****P < 0.0001 based on Student’s t-test

To explore the broader applicability of TPNC in hematologic malignancies, we generated TPNC from leukapheresis samples of patients with ALL and AML and assessed their cytotoxic potential (Fig. 7A). Against established leukemia cell lines, including CCRF-CEM, MOLT-4, HL-60, and THP-1, TPNC exhibited cytotoxicity levels comparable to those of conventional CIK cells (Fig. 7B and C). However, when tested against primary leukemia cells obtained directly from ALL and AML patients, TPNC demonstrated significantly enhanced cytotoxic activity (Fig. 7D and E), highlighting their superior tumor-targeting capability in patient-derived settings.

Importantly, TPNC effectively eliminated leukemia cells derived from both the priming donor and unrelated patients, indicating cross-sample reactivity. These results suggest that TPNC possess broad cytotoxic potential across hematologic malignancies, warranting further investigation into donor–recipient compatibility and clinical feasibility.

To further validate the therapeutic potential of TPNC, we directly compared their cytotoxic activity against patient-derived ALL cells with that of anti-CD19 CAR-NK cells and primary NK (pNK) cells. Remarkably, TPNC demonstrated cytotoxicity that was not only comparable to, but in some cases exceeded, that of CAR-NK cells (Fig. 7F and G), underscoring their potency despite the absence of genetic engineering.

These data collectively support the clinical relevance of TPNC as a robust allogeneic cell therapy platform capable of targeting a range of hematologic malignancies without the need for antigen-specific genetic modification.

## Discussion

In this study, we developed a novel cytotoxic lymphocyte product TPNC by co-culturing CBMC with irradiated, patient-derived tumor cells under defined cytokine conditions. Without any genetic modification, TPNC acquired potent antitumor activity across RRMM, AML and ALL. These cells demonstrated robust cytotoxicity, inflammatory cytokine production, and tumor suppression in vivo, highlighting their potential as a broadly applicable cellular immunotherapy platform.

Recent advances in adoptive cellular therapies such as CAR-T cells, CIK cells, and memory-like NK cells have shown therapeutic promise in hematologic malignancies [30–33]. However, each approach presents inherent limitations. CAR-T cells offer antigen-specific precision but require autologous cell harvesting and genetic manipulation, which leads to logistical, safety, and manufacturing challenges [34–37]. CIK and NK cells can deliver rapid cytotoxicity without MHC-restriction, yet they frequently exhibit limited tumor selectivity and persistence [38–40]. Memory-like NK cells show characteristics of recall responses and non-MHC-mediated killing, but their antigenic specificity is mainly confined to viral or stress-induced ligands and lacks the clonal diversity observed in adaptive T cells [20, 41].

TPNC may overcome some of these challenges by combining the innate flexibility of NK cells with the antigen-influenced specificity of adaptive immune responses. Functionally, they operate through two distinct mechanisms of cytotoxicity: one relies on LFA-1 and DNAM-1 for non-MHC-mediated killing, and the other depends on TCR recognition restricted by MHC class I. This dual functionality was confirmed in MHC-blocking experiments. Blocking HLA class I did not affect TPNC-mediated lysis of established MM cell lines but significantly impaired cytotoxicity against autologous RRMM samples. This result suggests that TCR signaling becomes more critical in immunoevasive primary tumors. Supporting this, TCR repertoire analysis showed a restricted clonal expansion with skewed TRBV usage, consistent with antigen-driven selection. Furthermore, cytotoxic function was lost when APCs were removed during priming, indicating that effective programming requires functional antigen presentation.

Beyond shaping the TCR repertoire, antigen-specific priming also appeared to influence effector programming. TPNC expressed elevated levels of perforin, granzyme B, and LFA-1 compared with CIK cells, despite both populations sharing the CD3⁺CD8⁺CD56⁺ phenotype. This functional distinction likely reflects their modes of generation. CIK cells are expanded through nonspecific CD3 crosslinking [42, 43], whereas TPNC are primed with patient-derived tumor cells that provide physiologic TCR ligands. Prior studies have shown that engagement of the TCR complex with its cognate antigen activates signaling pathways such as MAPK, PI3K–mTOR, and NFAT [44]. These pathways collectively enhance the production of cytotoxic granules, promote granule polarization, and increase LFA-1 clustering, ultimately facilitating more effective immune synapse formation and tumor cell killing [45–47]. This functional enhancement in TPNC is likely driven by both TCR-mediated signaling and the cytokine milieu present during priming. Specifically, IL-2 supports cell proliferation and the expression of cytolytic genes [48, 49]; IL-18 enhances IFN-γ production and degranulation [50, 51]; and IL-21 promotes sustained granule formation and metabolic activation via STAT3 and mTOR [52–54].

In addition to their role in effector programming, the cytokines used in our culture system may also have influenced the phenotypic identity of TPNC. The co-expression of CD3, CD8, and CD56 suggests the emergence of an NKT-like phenotype, which is not typical of conventional T or NK cells. Previous studies have shown that IL-18, particularly in combination with IL-12 or IL-15, can induce trained immunity in CD3⁺CD56⁺ T cells by enhancing their cytotoxicity and memory-like properties [55]. Similarly, IL-21 has been reported to promote the differentiation of memory CD8⁺ T cells with innate-like functional profiles, including rapid degranulation and the expression of NK-associated markers such as CD56 and NKG2D [27]. These findings suggest that the combined effects of IL-18 and IL-21 may have created a cytokine milieu capable of reprogramming CD8⁺ T cells toward an NKT-like phenotype, especially in the context of tumor antigen exposure and APC-mediated TCR engagement.

Together, these antigen- and cytokine-derived signaling likely contributed to a transcriptional and functional effector program uniquely suited for potent cytotoxicity. These mechanistic insights may also explain why CD8⁺ NKT-like cells, rather than NK cells, became the dominant population in our co-culture system.

The antigen specificity of TPNC was also shaped by the tumor used for priming. TPNC generated from RRMM, ALL, or AML patients were unable to kill tumor cells from unrelated cancer types, suggesting that their TCR repertoire was shaped by the antigenic landscape of the original malignancy (Fig. S7). However, within each malignancy subtype, TPNC exhibited cross-reactivity across unrelated patient samples. This supports the existence of conserved tumor-associated antigens within disease subtypes and highlights the potential for semi-personalized donor–target matching guided by shared neoantigens or clonal mutation profiles.

In vivo studies further support the translational potential of TPNC. In mouse xenograft models, TPNC treatment led to tumor suppression and peripheral persistence without evidence of off-target cytotoxicity or GvHD-like symptoms, as confirmed by body weight monitoring and histological evaluation. These findings suggest that TPNC may serve as a safe and effective allogeneic immunotherapy. Because they are derived from cord blood, require no genetic engineering, and can be prepared in advance, TPNC offer a potentially accessible treatment strategy for relapsed and refractory hematologic malignancies.

Nonetheless, several important challenges remain. The specific tumor antigens recognized by TPNC have not yet been identified. Determining these targets will be crucial for improving donor matching and therapeutic precision. In addition, the use of immunodeficient mouse models restricts the evaluation of memory formation, long-term persistence, and interactions with the host immune system. Moreover, the limited availability of primary tumor cells from RRMM patients posed a significant barrier to generating patient-derived xenograft (PDX) models alongside TPNC production. Although attempted in preliminary experiments, engraftment efficiency was low, likely due to insufficient tumor burden and cell number. Finally, the current reliance on patient-derived tumor feeder cells may hinder GMP-compliant manufacturing processes. Additionally, the safety and efficacy of TPNC in other hematologic malignancies beyond RRMM will require further investigation to address potential differences in antigen recognition and off-target effects. Developing artificial feeder systems or using antigen-loaded APCs could help standardize production and facilitate clinical translation.

## Conclusion

TPNC are non-genetically engineered cytotoxic lymphocytes that integrate innate and adaptive mechanisms. With dual recognition pathways, durable activity, favorable safety, and scalable production, they offer a clinically relevant platform for immunotherapy in relapsed and refractory hematologic malignancies.

## Supplementary Information

Below is the link to the electronic supplementary material.

Supplementary Material 1

Supplementary Material 2

Supplementary Material 3

## Data Availability

No datasets were generated or analysed during the current study.

## Abbreviations

ALL: Acute lymphoblastic leukemia
AML: Acute myeloid leukemia
ANOVA: Analysis of variance
APC: Antigen-presenting cell
aCD19 CAR: Anti-CD19 chimeric antigen receptor
BLI: Bioluminescence imaging
CAR: Chimeric antigen receptor
CBMC: Cord blood mononuclear cells
CDR3: Complementarity-determining region 3
CIK: Cytokine-induced killer
DC: Dendritic cell
DNAM-1: DNAX accessory molecule-1
FACS: Fluorescence-activated cell sorting
GvHD: Graft-versus-host disease
HLA: Human leukocyte antigen
HPL: Human platelet lysate
ICAM: Intercellular adhesion molecule
IFN-γ: Interferon-gamma
IL-2: Interleukin-2
IL-18: Interleukin-18
IL-21: Interleukin-21
LFA-1: Lymphocyte function-associated antigen-1
MFI: Mean fluorescence intensity
MHC: Major histocompatibility complex
PBMC: Peripheral blood mononuclear cell
PBS: Phosphate-buffered saline
pNK: Primary natural killer cell
RRMM: Relapsed or refractory multiple myeloma
SD: Standard deviation
TCR: T cell receptor
TNF-α: Tumor necrosis factor-alpha
TPNC: Tumor-priming CD8⁺ NKT-like cell
TRBV: T cell receptor beta variable region
