# Supplementary material for: Tumor-priming CD8+ natural killer T-like cells as an efficient novel cell therapy for relapsed/refractory multiple myeloma
Source: Exp Hematol Oncol. 2025 Sep 29;14:116. doi: 10.1186/s40164-025-00707-7 (PMC12482268; doi:10.1186/s40164-025-00707-7)
Supplement: Supplementary file 3 — Supplementary Material 3 [file 40164_2025_707_MOESM3_ESM.docx]

**Tumor-priming CD8^+^ natural killer T-like cells as an efficient novel cell therapy for relapsed/refractory multiple myeloma**

**Supplementary Materials and Methods**

**Live cell imaging**

Single cancer cell arrays were fabricated using IM-9 cells, as previously described (1), and were used to quantitatively analyze TPNC-cancer- and CIK-cancer cell interactions. A modified Olympus IX 83 epi-fluorescence microscope with a 40 × (UPlanFLN, NA = 1.30) objective lens and an ANDOR Zyla 4.2 sCOMS camera were used for the imaging experiments. The microscope was automatically controlled using Micro-manager. The microscope stage was equipped with a Chamlide TC incubator system (Live Cell Instrument) maintaining cell culture conditions (37°C, CO_2_ 5%). The acquired images were processed using the ImageJ software. CIK cells and TPNC were stained with 1 μg/mL of LysoTracker Deep Red (Invitrogen, Carlsbad, CA, USA) by incubating at 37℃ for 1 h. IM-9 cells were labeled with 10 μg/mL CellTrace CFSE (Invitrogen) by incubating at 37℃ for 15 min prior to the fabrication of the single cell arrays. LysoTracker-labeled effector cells in complete RPMI-1640 medium containing 10 μg/mL propidium iodide (PI) were seeded onto a single-cell array of IM-9 cells loaded in a magnetic chamber. The effector cell-seeded chamber was mounted on a microscope stage equipped with an incubator system, and time-lapse imaging was performed at 3-min intervals for 3 h.

**NK cell culture and anti-CD19 CAR-NK cell generation**

Cord blood-derived mononuclear cells were isolated by density gradient centrifugation and cultured in CTS™ NK-Xpander™ medium (Gibco, USA) supplemented with IL-2, IL-18, and IL-21 on day 0. On days 3 and 5, only IL-2 was replenished to support NK cell expansion. For CAR-NK cell generation, cells were seeded into 24-well plates on day 7 and transduced the following day with a lentiviral vector encoding an anti-CD19 CAR construct containing 4-1BB and CD3ζ signaling domains. Transduction was performed using spinoculation in the presence of polybrene and the TBK1 inhibitor BX795. CAR-NK cells were then expanded for 7–10 days before use in functional assays. For non-transduced NK cell culture, IL-2 and IL-21 were supplemented every 2–3 days after day 7 to maintain NK cell viability and activity.

**Magnetic isolation of CD3⁺ cells**

CD3⁺ T cells were isolated from 1 × 10⁸ cord blood mononuclear cells (CBMC) using anti-CD3 MicroBeads (Miltenyi Biotec, Cat# 130-050-101) and LS columns (Miltenyi Biotec, Cat# 130-042-401), according to the manufacturer’s instructions.

**Reference**

1. Kim SE, Kim H, Doh J. Single cell arrays of hematological cancer cells for assessment of lymphocyte cytotoxicity dynamics, serial killing, and extracellular molecules. Lab Chip. 2019;19(11):2009-18.

**Supplementary Figures**

**Figure S1**

**
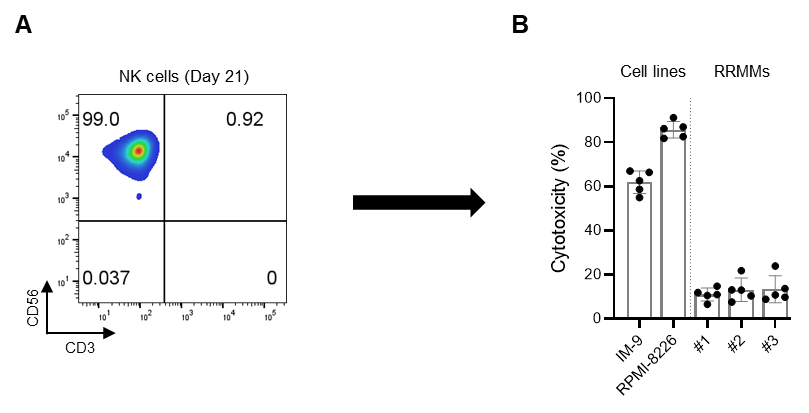
**

**Supplementary Figure S1. Cytotoxicity of cord blood derived-NK cells against MM cell lines and RRMM samples**

**(A)** NK cells were cultured for 21 days and analyzed by FACS to confirm CD3⁻CD56⁺ identity. **(B)** NK cells were co-cultured with MM cell lines (IM-9, RPMI-8226) and RRMM patient-derived samples (#1–3) at an effector-to-target ratio of 5:1 for 4 hours. Cytotoxicity was quantified based on CFSE⁺FVD⁺ target cell death. Data are presented as mean ± SD of five independent experiments.

**Figure S2**

**
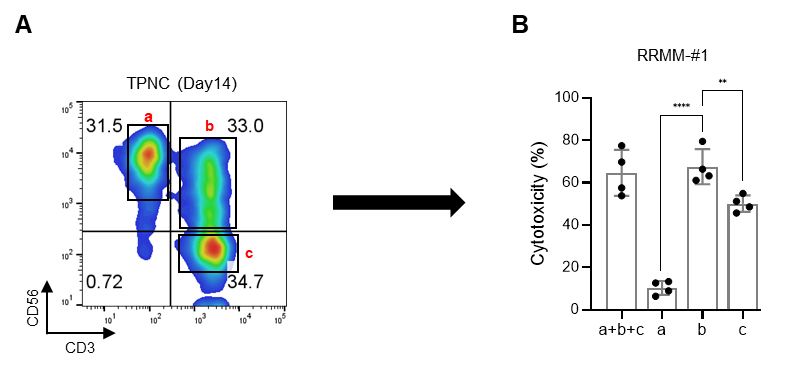
**

**Supplementary Figure S2. Subset analysis of TPNC cytotoxicity**

**(A)** TPNC were analyzed at day 14 for population distribution using flow cytometry. **(B)** Each subset (a: CD3⁻CD56⁺, b: CD3⁺CD56⁺, c: CD3⁺CD56⁻) was sorted and tested for cytotoxicity against RRMM cells. Cytotoxicity was measured at a 5:1 effector-to-target (E:T) ratio using CFSE⁺FVD⁺ markers after a 4 hour co-culture. Data are presented as mean ± SD of four independent experiments. ** *P* <0.01; **** *P* < 0.0001 based on Student’s t-test.

**Figure S3**

**
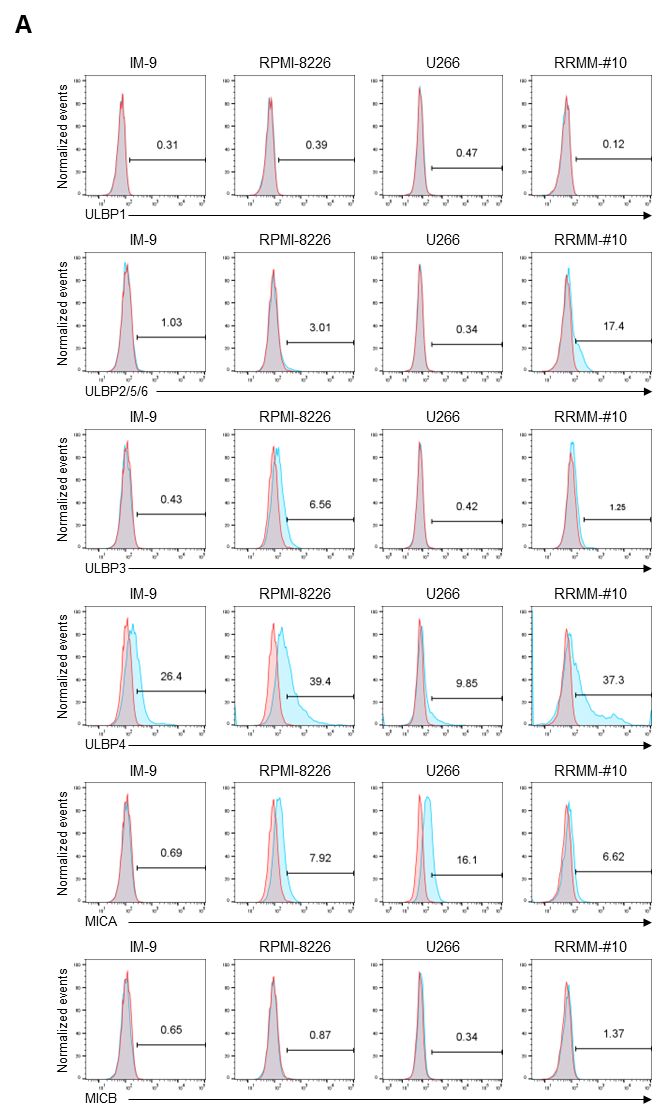
**

**Supplementary Figure S3. Expression of NKG2D ligands on MM cell lines and RRMM cells**

Expression of NKG2D ligands was assessed by flow cytometry in multiple myeloma (MM) cell lines (IM-9, RPMI-8226, U266) and a primary RRMM sample (RRMM-#10). Surface levels of ULBP1, ULBP2/5/6, ULBP3, ULBP4, MICA, and MICB were evaluated. Histograms represent normalized fluorescence intensity; red lines indicate isotype controls and blue lines represent ligand-stained samples.

**Figure S4**


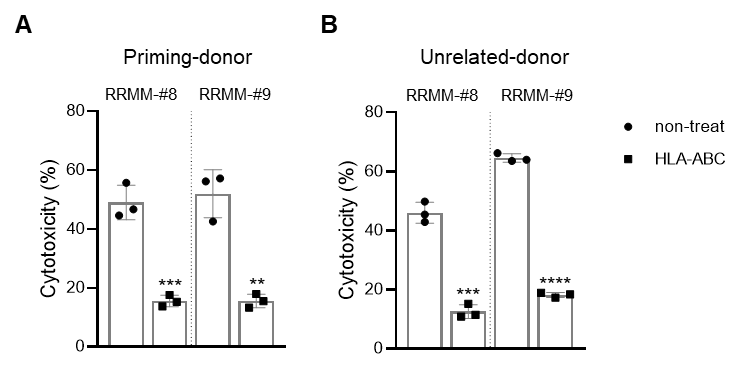


**Supplementary Figure S4. HLA class I blockade reduces TPNC-mediated cytotoxicity against both autologous and unrelated RRMM samples**

TPNC were co-cultured with RRMM target cells in the presence or absence of HLA-ABC blocking antibody. (A) Cytotoxicity against autologous (priming donor) targets: TPNC were generated from RRMM-#8 or RRMM-#9 and tested against their respective autologous targets. (B) Cytotoxicity against unrelated targets: TPNC generated from RRMM-#10 were tested against unrelated RRMM-#8 and RRMM-#9 target cells. Cytotoxicity was measured at a 5:1 effector-to-target (E:T) ratio using CFSE⁺FVD⁺ markers after a 4-hour co-culture. Data are presented as mean ± SD of three independent experiments. ** *P* < 0.01, *** *P* < 0.001, **** *P* < 0.0001 based on Student’s t-test.

**Figure S5**


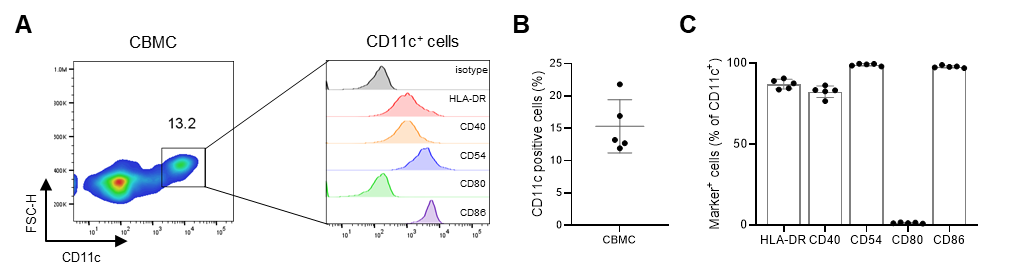


**Supplementary Figure S5. Phenotypic characterization of CD11c⁺ APCs in CBMC**

**(A)** Representative flow cytometry plots showing the presence of CD11c⁺ cells (~13.2%) within CBMC. Histograms depict surface expression of APC-associated markers (HLA-DR, CD40, CD54, CD80, CD86) within the CD11c⁺ population. **(B)** Frequency of CD11c⁺ cells as a percentage of total CBMC (n = 5). **(C)** Expression levels of APC-associated markers among CD11c⁺ cells. Data are presented as mean ± SD of five independent experiments.

**Figure S6**

**
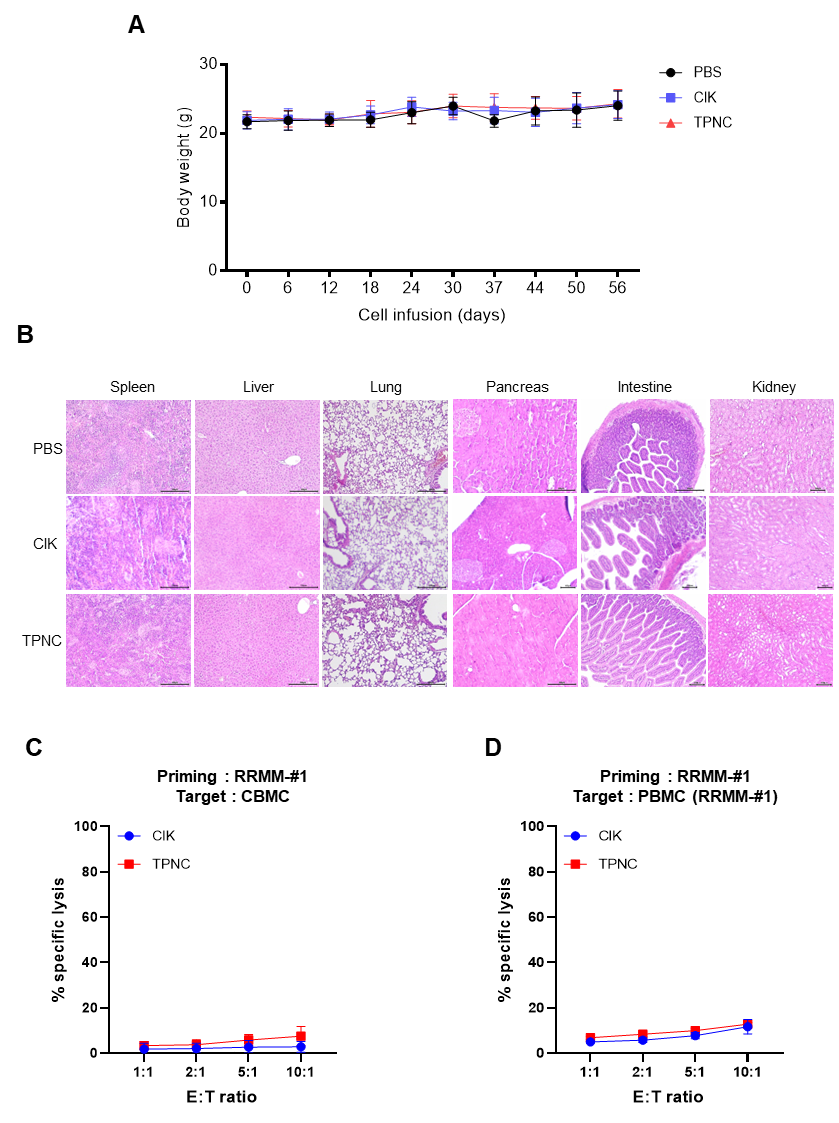
**

**Supplementary Figure S6. Safety evaluation of TPNC therapy**

TPNC and CIK cells were administered intravenously for 3 weeks at a dose of 1 × 10^8^ cells once a week, and the mice were sacrificed after 8 weeks. **(A)** Body weight changes of mice receiving different treatment. **(B)** Representative image of H&E staining for mice organs. **(C)** Cytotoxicity of TPNC and CIK against allogeneic CBMC **(D)** Cytotoxicity of TPNC and CIK against PBMC from RRMM-#1. Data are presented as mean ± SD of three independent experiments **(C, D)**.

**Figure S7**


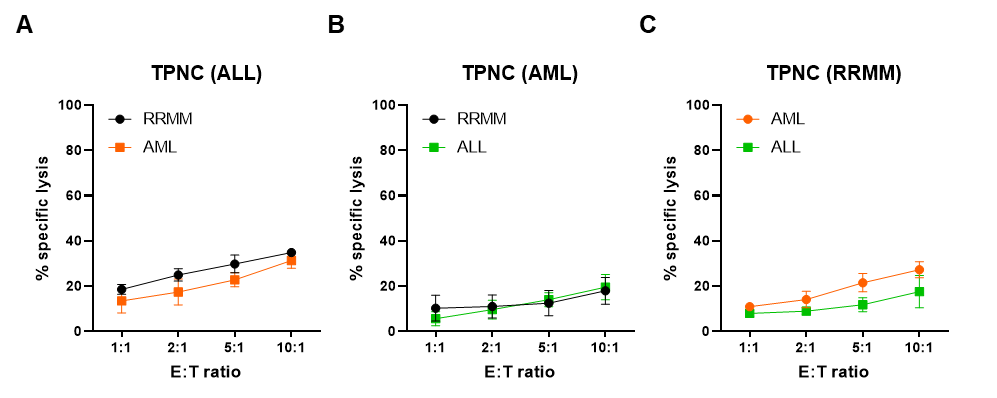


**Supplementary Figure S7**. **Tumor-specific cytotoxicity of TPNC against other hematologic malignancies**

Generate TPNC with various patient-derived cells (ALL, AML, RRMM) as feeder cells and analyze their cytotoxicity against different hematologic malignancies. Cells were co-cultured for 4 hours. Quantitative assessment of killed cells was performed in the CFSE^+^FVD^+^ region. **(A)** Cytotoxicity of TPNC (ALL) against RRMM and AML, **(B)** Cytotoxicity of TPNC (AML) against RRMM and ALL **(C)** Cytotoxicity of TPNC (RRMM) against AML and ALL. Data are presented as mean ± SD of three independent experiments **(A, B, C).**

**Table S1**

| UPN | Age, years | Sex | MM type | R-ISS | BM clonal plasma cells at the time of sample collection | Precvious treatments |
| --- | --- | --- | --- | --- | --- | --- |
| #1 | 73 | M | IgG, kappa | II | 100.00% | VAD - Auto - Vd - Rd - Pd - Daratumumab |
| #2 | 74 | M | LCD, kappa | III | 44.80% | VMP -Rd - Kd- Pd - Daratumumab - CTd - selinexor |
| #3 | 62 | M | IgG, kappa | II | 24.20% | VTd - Auto - KRd |
| #4 | 83 | M | IgA, lambda | III | 19.20% | Rd |
| #5 | 62 | M | IgG, kappa | II | 8.20% | VTd - Auto - KRd |
| #6 | 73 | F | LCD, lambda | III | 3.55% | Cd-Vd-Auto-Rd-CTd-Kd-Daratumumab-PCd |
| #7 | 63 | F | LCD, kappa | III | 34% | VTd |
| #8 | 68 | M | IgG, lambda | II | 3.52% | VTd-Auto-IRd |
| #9 | 66 | F | IgG, lambda | I | 5.47% | Td-Auto-Rd-Kd |
| #10 | 62 | F | LCD, lambda | II | 54.60% | VRd-Auto-Vd-Pd-Kd-Daratumumab |

**Supplementary Table S1. Clinical characteristics of the analyzed relapsed and refractory multiple myeloma (RRMM) patients.** Samples from RRMM patients were used for tumor-priming CD8^+^ natural killer T-like cells experiments. Abbreviations, UPN, unique patient number; M, male; F, female; R-ISS, revised international staging system; BM, bone marrow; LCD, light chain disease, IgG, immunoglobulin G, IgA, immunoglobulin A; VMP, bortezomib-melphalan-prednisolone; Rd, lenalidomide-dexamethasone; Kd, carfilzomib-dexamethasone; Pd, pomalidomide-dexamethasone; CTd, cyclophosphamide-thalidomide-dexamethasone; VAD, vincristine-doxorubicin-dexamethasone; Auto, autologous stem cell transplantation; Vd, bortezomib-dexamethasone; VTd, bortezomib-thalidomide-dexamethasone; KRd, carfilzomib-lenalidomide-dexamethasone; Cd, cyclophosphamide-dexamethasone; PCd, pomalidomide-cyclophosphamide-dexamethasone; IRd, ixazomib-lenalidomide-dexamethasone; Td, thalidomide-dexamethasone; DVTd, daratumumab-bortezomib-thalidomide-dexamethasone; VRd, bortezomib-lenalidomide-dexamethasone.

**Table S2**

| UPN | Age, years | Sex | Diagnosis | Cytogenetic and molecular risk groups (specific alterations) | Immunophenotyping | WBC at the time of sample collection (/μL) |
| --- | --- | --- | --- | --- | --- | --- |
| #1 | 68 | M | AML | Intermediate  (Trisomy21, *FLT3, DNMT3A, EZH2, NRAS, ZRSR2* ) | CD13, CD33, CD117, HLA-DR, CD71, Cytoplasmic MPO | 122,650 |
| #2 | 53 | F | AML | Intermediate | CD11c, CD33, CD64, CD117, CD56, Cytoplasmic MPO, CD71 | 163,410 |
| #1 | 54 | M | ALL | Poor (*BCR-ABL1*) | CD13, CD33, HLA-DR, TDT, CD19, CD34, Cytoplasmic CD79a | 461,400 |
| #2 | 60 | M | ALL | Poor (*BCR-ABL1*, p16) | CD13, CD10, CD19, CD20, HLA-DR, CD71, Cytoplasmic CD79a | 295,140 |

# Supplementary Table S2. Clinical characteristics of the analyzed newly diagnosed acute lymphoblastic leukemia (ALL) and acute myeloid leukemia (AML) patients. Samples from ALL and AML patients were used for tumor-priming CD8^+^ natural killer T-like cells experiments. Abbreviations, UPN, unique patient number; M, male; F, female; FLT3, FMS‐like tyrosine kinase 3; DNMT3A, DNA (cytosine-5)-methyltransferase 3; EZH2, Enhancer of zeste homolog 2;  NRAS,  NRAS proto-oncogene; ZRSR2, zinc finger CCCH-type, RNA binding motif and serine/arginine rich 2; BCR-ABL1, breakpoint cluster region protein-ABL Proto-Oncogene 1, Non-Receptor Tyrosine Kinase; HLA-DR,  major histocompatibility complex, class II, DR alpha; MPO, Myeloperoxidase; TDT, Terminal deoxynucleotidyl transferase; WBC, white blood cell.

**Table S3**

| **Antibody Name** | **Catalog Number** | **Manufacturer** |
| --- | --- | --- |
| anti-CD3-FITC | 561807 | BD Biosciences |
| anti-CD3-PE | 561803 | BD Biosciences |
| anti-CD56-PE-cy7 | 335791 | BD Biosciences |
| anti-CD4-PE | 555347 | BD Biosciences |
| anti-CD8-PE | 555367 | BD Biosciences |
| anti-CD16-PE | 556619 | BD Biosciences |
| anti-CD62L-PE | 555544 | BD Biosciences |
| anti-CD45RA-FITC | 347723 | BD Biosciences |
| anti-CD45RO-PE | 12-0457-42 | eBioscience^™^ |
| anti-CD57-PE | 560844 | BD Biosciences |
| anti-NKp30-PE | 558407 | BD Biosciences |
| anti-NKp44-PE | 558563 | BD Biosciences |
| anti-NKp46-PE | 557991 | BD Biosciences |
| anti-NKp80-PE | 566329 | BD Biosciences |
| anti-NKG2C-PE | FAB138P | R&D Systems |
| anti-NKG2D-PE | 557940 | BD Biosciences |
| anti-CD69-PE | 555531 | BD Biosciences |
| anti-NKG2A-PE | FAB1059P | R&D Systems |
| anti-CD158b-PE | 559785 | BD Biosciences |
| anti-TIGIT-PE | 372704 | BioLegend |
| anti-GITR-PE | 311604 | BioLegend |
| anti-BTLA-PE | 558485 | BD Biosciences |
| anti-LAG3-PE | 565616 | BD Biosciences |
| anti-CXCR3-PE | 353706 | BioLegend |
| anti-CXCR4-PE | 306506 | BioLegend |
| anti-CXCR6-PE | 356004 | BioLegend |
| anti-Perforin-FITC | 308103 | BioLegend |
| anti-Granzyme B-FITC | 515403 | BioLegend |
| anti-CD45-FITC | 368507 | BioLegend |
| anti-FMC63 antibody | FM3-Y45-25tests | ACRObiosystems |
| anti-mouse IgG1-PE-cy7 | 406613 | BioLegend |
| anti-ULBP1-PerCP | FAB1380C | R&D Systems |
| anti-ULBP2/5/6-PE | FAB1298P | R&D Systems |
| anti-ULBP3-PE | FAB1517P | R&D Systems |
| anti-ULBP4-PE | FAB6285P | R&D Systems |
| anti-MICA-PE | FAB1300P | R&D Systems |
| anti-MICB-PE | FAB1599P | R&D Systems |
| anti-CD11c-PC5 | IM3707 | Beckman Coulter |
| anti-HLA-DR-PE | IM1639 | Beckman Coulter |
| anti-CD40-PE | IM1936U | Beckman Coulter |
| anti-CD54-PE | IM1239U | Beckman Coulter |
| anti-CD80-PE | PN IM1976U | Beckman Coulter |
| anti-CD86-PE | IM2729U | Beckman Coulter |
| anti-LFA-1-PE | 363405 | BioLegend |
| anti-DNAM-1-PE | 337106 | BioLegend |
| anti-CD112-PE | 337409 | BioLegend |
| anti-CD155-FITC | 337628 | BioLegend |
| anti-ICAM-1-PE | 353105 | BioLegend |
| anti-ICAM-2-PE | 328505 | BioLegend |
| anti-ICAM-3-PE | 3330005 | BioLegend |
| LFA-1 blocker | 301202 | BioLegend |
| DNAM-1 blocker | 338302 | BioLegend |
| HLA-ABC blocker | 311402 | BioLegend |

**Supplementary Table S3. Antibodies used in flow cytometry studies**

FITC, fluorescein isothiocyanate; PE, phycoerythrin; PE-Cy7, phycoerythrin-cyanine7; PerCP, peridinin-chlorophyll protein complex; PC5, phycoerythrin-cyanine5

**Supplementary Movie S1 Legend**

Live imaging of interaction between human TPNC/CIK cells (yellow border line), IM-9 cells (Green) and PI (Blue) using fluorescence microscopy.
